# Pregnancy, Proteinuria, Plant-Based Supplemented Diets and Focal Segmental Glomerulosclerosis: A Report on Three Cases and Critical Appraisal of the Literature

**DOI:** 10.3390/nu9070770

**Published:** 2017-07-19

**Authors:** Rossella Attini, Filomena Leone, Benedetta Montersino, Federica Fassio, Fosca Minelli, Loredana Colla, Maura Rossetti, Cristiana Rollino, Maria Grazia Alemanno, Antonella Barreca, Tullia Todros, Giorgina Barbara Piccoli

**Affiliations:** 1 Materno-Foetal Unit, Department of Surgery, University of Torino, 10100 Turin, Italy; rossella.attini@gmail.com (R.A.); filomena.leone@unito.it (F.L.); benedettamontersino@yahoo.it (B.M.); federica.fassio@hotmail.it (F.F.); minellifosca@hotmail.it (F.M.); mg.allemanno@inwind.it (M.G.A.); tullia.todros@unito.it (T.T.); 2 SCDU Nephrology, Città della Salute e della Scienza, University of Torino, 10100 Turin, Italy; lcolla@cittadellasalute.to.it (L.C.); mrossetti2@cittadellasalute.to.it (M.R.); 3 SCDU Nephrology, Giovanni Bosco Hospital, University of Torino, 10100 Turin, Italy; cristiana.rollino@libero.it; 4 Department of Medical Sciences, University of Torino, 10100 Turin, Italy; antonella.barreca@libero.it; 5 Department of Biological and Clinical Sciences, University of Torino, 10100 Turin, Italy; 6 Nephrology, Centre Hospitalier Le Mans, 72000 Le Mans, France

**Keywords:** vegan diet, plant-based diet, proteinuria, chronic kidney disease, focal segmental glomerulosclerosis, pregnancy, preterm delivery, hyperfiltration, low protein diets

## Abstract

Chronic kidney disease (CKD) is increasingly recognized in pregnant patients. Three characteristics are associated with a risk of preterm delivery or small for gestational age babies; kidney function reduction, hypertension, and proteinuria. In pregnancy, the anti-proteinuric agents (ACE–angiotensin converting enzyme-inhibitors or ARBS -angiotensin receptor blockers) have to be discontinued for their potential teratogenicity, and there is no validated approach to control proteinuria. Furthermore, proteinuria usually increases as an effect of therapeutic changes and pregnancy-induced hyperfiltration. Based on a favourable effect of low-protein diets on proteinuria and advanced CKD, our group developed a moderately protein-restricted vegan-vegetarian diet tsupplemented with ketoacids and aminoacids for pregnant patients. This report describes the results obtained in three pregnant patients with normal renal function, nephrotic or sub-nephrotic proteinuria, and biopsy proven diagnosis of focal segmental glomerulosclerosis, a renal lesion in which hyperfiltration is considered of pivotal importance (case 1: GFR (glomerular filtration rate): 103 mL/min; proteinuria 2.1 g/day; albumin 3.2 g/dL; case 2: GFR 86 mL/min, proteinuria 3.03 g/day, albumin 3.4 g/dL; case 3: GFR 142 mL/min, proteinuria 6.3 g/day, albumin 3.23 g/dL). The moderately restricted diet allowed a stabilisation of proteinuria in two cases and a decrease in one. No significant changes in serum creatinine and serum albumin were observed. The three babies were born at term (38 weeks + 3 days, female, weight 3180 g-62th centile; 38 weeks + 2 days, female, weight 3300 g-75th centile; male, 38 weeks + 1 day; 2770 g-8th centile), thus reassuring us of the safety of the diet. In summary, based on these three cases studies and a review of the literature, we suggest that a moderately protein-restricted, supplemented, plant-based diet might contribute to controlling proteinuria in pregnant CKD women with focal segmental glomerulosclerosis. However further studies are warranted to confirm the potential value of such a treatment strategy.

## 1. Background

Chronic kidney disease (CKD) is increasingly diagnosed in pregnant patients, mainly as a result of greater attention to this emerging problem, and also thanks to the good results obtained in severe CKD up to the dialysis phase [1,2,3,4,5,6]. Furthermore, pre-natal care is an increasingly recognised opportunity for the early diagnosis of potentially treatable CKD through a different diagnosis from preeclampsia [7,8,9,10,11,12].

The most common pregnancy-related adverse events in CKD patients are pre-term delivery, intrauterine growth restriction, and the progression of maternal kidney disease [1,2,5,13].

Three characteristics of CKD are associated with a higher risk; kidney function reduction, hypertension, and proteinuria. The risk is inversely related to baseline kidney function [2,14,15]. However, the excellent results obtained with intensive dialysis in pregnancy underline the possibility of modifying the clinical outcome even in patients with the most severe conditions [3,16].

Baseline hypertension is the second element with a detrimental effect on pregnancy outcomes, and its importance is probably greater in patients with CKD [17,18,19,20]. While the target blood pressure levels at which hypertensive patients should aim in pregnancy is still a matter of controversy, the stabilization or normalization of blood pressure has been associated with a decreased risk of adverse outcomes [17,19,20,21].

Proteinuria represents the third main element associated with adverse outcomes, once more with a cumulative and possibly multiplicative effect if combined with hypertension and renal function impairment [2,4,22]. In spite of the growing body of evidence on the relationship between proteinuria and pregnancy outcomes, no therapeutic approach has so far been identified to control it in pregnancy, and widely used reno-protective and anti-proteinuric agents, including (angiotensin converting enzyme) ACE inhibitors and angiotensin receptor inhibitors, are banned in pregnancy due to their suspected teratogenicity [23,24,25].

Based on a series of small-scale studies reporting on a favourable effect of low-protein diets on proteinuria, our group developed a moderately protein-restricted vegan-vegetarian diet, supplemented with ketoacids and aminoacids, for pregnant patients with renal function impairment, advanced diabetic nephropathy, or severe proteinuria [26,27,28,29,30].

To try to better understand the effect of the diet on proteinuria, we focused this report on three patients with normal renal function and biopsy proven diagnosis of focal segmental glomerulosclerosis (FSGS), a renal lesion, for which the interpretation is undergoing extensive reevaluation and in which hyperfiltration is considered of pivotal importance [31,32,33,34,35].

## 2. The Cases

### 2.1. Case 1

A Caucasian woman, 42 years old at the time of the present report, 38 years old at the start of pregnancy, had developed a full blown nephrotic syndrome at 17 years of age, which was diagnosed as focal segmental glomerulosclerosis (FSGS); at diagnosis, her kidney function was normal, and proteinuria ranged between 9 and 11 g/day. She was treated with different combinations of steroids and immunodepressive agents, including cyclophosphamide, cyclosporine A, and mycophenolate mophetile; indomethacin had also been employed for one year as an antiproteinuric agent. Proteinuria only partially responded and, on average, ranged from 2 to 5 g/day. In 1999 and 2000, the patient had experienced two early spontaneous miscarriages (in both cases at the ninth gestational week).

From 2008 to 2011, she was treated with mycophenolate mophetile, and she re-converted to cyclosporine A in 2011, when she decided to try again to have a baby. While renal function was always normal, proteinuria remained in the subnephrotic-nephrotic range (2 to 4 g/day) until pregnancy.

The following were the results of the last laboratory tests before pregnancy: serum creatinine 0.6 mg/dL; creatinine clearance on 24 h urine collection: 103 mL/min; proteinuria 2.1 g/day; serum albumin 3.2 g/dL. She was on treatment with ACE inhibitors and angiotensin receptor blockers (ARBS) (Telmisartan and Ramipril, both discontinued at positive pregnancy test) and cyclosporine A 100 mg/day.

She was referred to the unit dedicated to kidney and pregnancy at the sixth gestational week; a low-protein supplemented diet (diet 1, reported in Appendix A) was started at the 13th gestational week.

**Figure 1:**
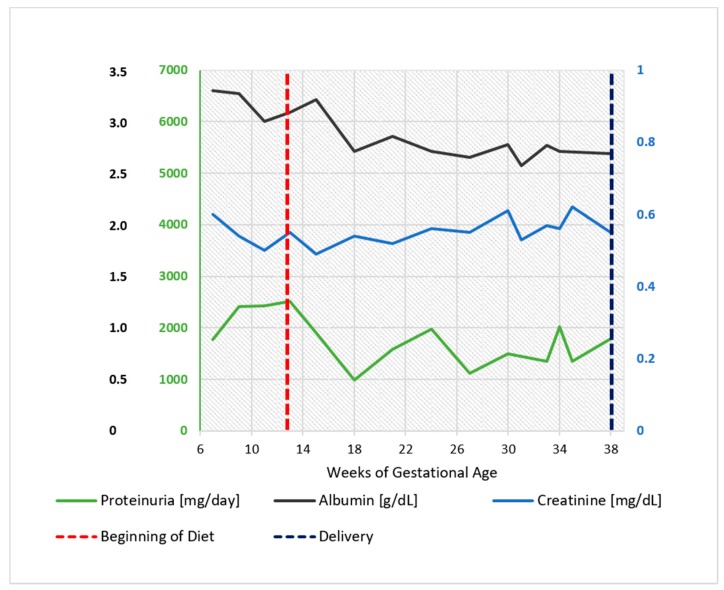
Case 1. Serum albumin, serum creatinine, and 24 h proteinuria during pregnancy and before and after the start of the diet. Note: serum creatinine minimum: 0.55 mg/dL, maximum 0.7 mg/dL.

As shown in Figure 1, proteinuria, in the range of 2 to 4 g/day under treatment with ACE inhibitors and ARBS, did not increase after the start of the diet and displayed an even lower range throughout her pregnancy (1 to 2 g/day) (Figure 1).

The patient was treated with Acetylsalicylic acid (75 mg/day) from the seventh gestational week; Alpha-Methyldopa was used from the ninth gestational week to control hypertension (maximum dosage of 1 g/day at term).

**Figure 2:**
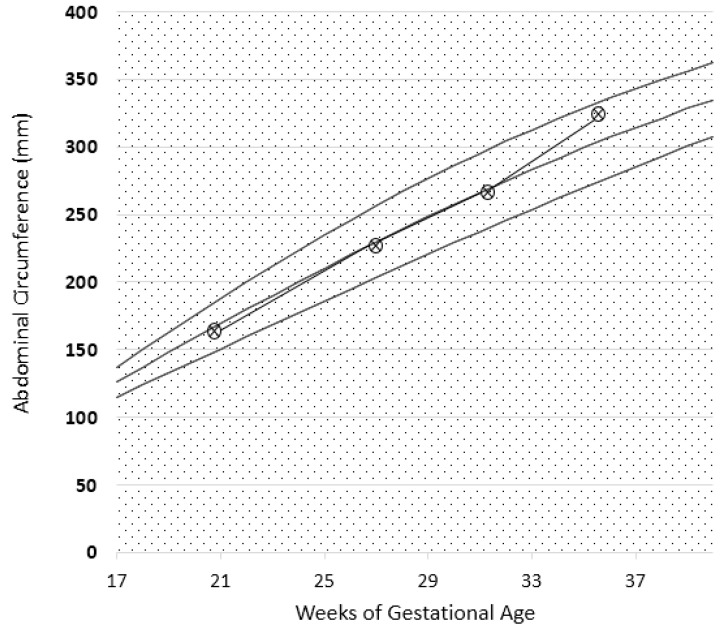
Intrauterine growth curve in Case 1. The parameter analysed is the abdominal circumference (CA). Report based on SIEOG (Italian Society of Obstetric-Gynecological Ecography) Guidelines. Biometric curves: Paladini et al.

Fetal growth, as well as uterine and umbilical arterial Doppler, were normal, as reported in Figure 2. A female baby, normal for her gestational age was delivered at 38 weeks and three days of ultrasound-confirmed gestational age (weight 3180 g, Apgar index 9 at the 1st min, 9 at the 5th min; neonatal weight was in the 62th centile of the Italian neonatal weight curves (Ines Charts)).

**Figure 3:**
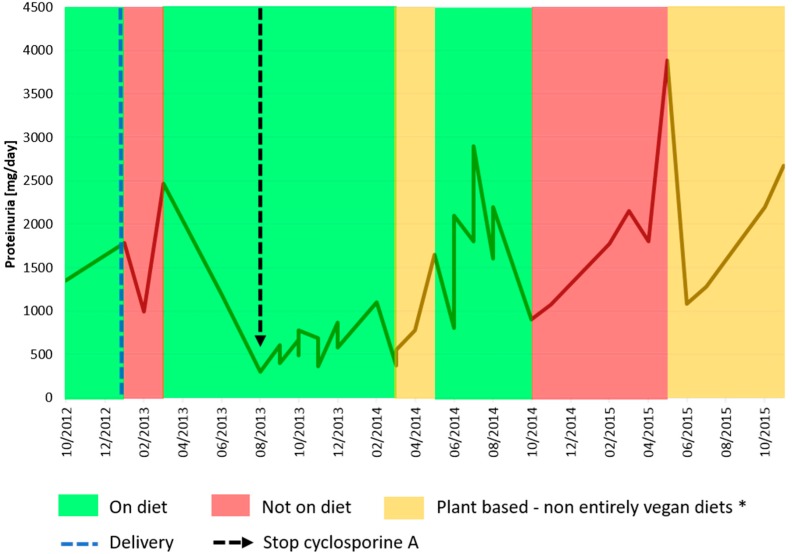
24 h-proteinuria pattern after pregnancy. * From March to May 2014, the patient observed a Mediterranean weight loss diet; since May of 2015, the patient has followed a normocaloric plant-based diet, avoiding dairy products.

Immediately after delivery, the patient discontinued the low-protein diet, leading to an increase in proteinuria. She resumed the diet three months later and, for the first time since the onset of her disease, proteinuria consistently decreased below <500 mg/day, allowing discontinuation of cyclosporine A (Figure 3). Over the following four years, she resumed a plant-based diet twice, however only once for a relatively long period, overall suggesting some correspondence between proteinuria and dietary patterns, with a nadir of proteinuria following a four to eight week the change in diet (Figure 3). She dropped out again from follow-up for one year; at the time of the present report, her clinical conditions are good, her kidney function is normal, and the patient is motivated to resume a plant-based supplemented diet. The child is in good health, with normal physical and psychological development.

### 2.2. Case 2

**Figure 4:**
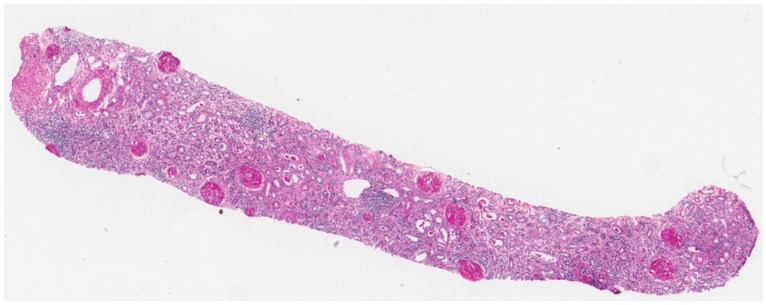
First kidney biopsy in Case 2. Several glomeruli with global sclerosis and moderate inflammatory interstitial infiltrate (A: PAS-Schiff’s periodic acid-original magnification 20×).

A Caucasian woman, 28 years old at the time of the present report, underwent a kidney biopsy at 18 years of age for a full-blown nephrotic syndrome with abnormal renal function. The kidney biopsy identified membranous nephropathy associated with florid epithelial crescents in five glomeruli, occupying from 5% to 90% of Bowman’s space; more than half of the glomeruli were sclerotic (23 glomeruli, 18 with global sclerosis, Figure 4). Limited segments of the glomerular basement membranes were moderately thickened and showed subepithelial deposits in PTAH (Phosphotungstic Acid-Hematoxylin) and AFOG (Acidic Fuchsin Orange G coloration) stains. Immunofluorescent analysis revealed granular immunoglobulin IgG (3+), C3 (3+), C4 (1+), and C1q (1+) deposition along the glomerular capillary wall. She underwent treatment with cyclophosphamide, plasma exchanges, and steroids, followed by steroids and cyclosporine A. While her kidney function returned to the normal range, subnephrotic proteinuria persisted and she underwent a second kidney biopsy four years later. At that time, the antibodies anti PlA2r tested negative and kidney function was normal.

**Figure 5:**
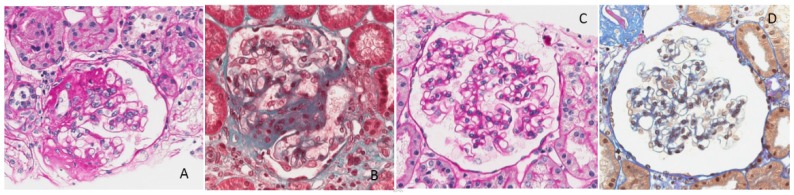
Second kidney biopsy in Case 2. (**A**,**B**) Foci of segmental sclero-hyalinosis with obliteration of the glomerular tuft by increased matrix and hyaline material. Sclerotic lesions are localized in perihilar and/or peripheral segments and form adhesions to Bowman’s capsule. There is no obvious podocyte hypertrophy or hyperplasia; (**A**) PAS original magnification 400×; (**B**) Masson trichrome original magnification 400×; (**C**,**D**) Capillary walls are no more thickened: AFOG (Acidic Fuchsin Orange G coloration) stain does not highlight protein deposits in glomerular basement membranes; (**C**) PAS original magnification 200×; (**D**) AFOG original magnification 400×.

The biopsy did not identify the previously reported membranous lesions but found signs of focal segmental glomerular sclerosis, which was considered a secondary effect of nephronic reduction due to the previous disease (kidney biopsy with 27 glomeruli, 12 of these with global sclerosis, and three with segmental sclerotic lesions) Immunofluorescent analysis did not reveal the previous deposits of IgG and C3 (Figure 5A–D).

A further kidney biopsy was performed two years later in the presence of nephrotic proteinuria, suggesting a relapse of membranous lesions with granular subepithelial capillary wall deposits of IgG (3+) C3 (3+), IgM (++), and C1q (1+); six out of ten glomeruli displayed global sclerosis. She was treated with steroids and cyclophosphamide, in accordance with the Ponticelli scheme and with Rituximab, followed by cyclosporine A.

At the start of her pregnancy, her treatment consisted of 125 mg/day of cyclosporine A and 75 mg/day of acetylsalicylic acid; an ACE inhibitor (Ramipril) was discontinued at a positive pregnancy test. Her biochemical data at referral were as follows: creatinine clearance 86 mL/min, serum creatinine 0.8 mg/dL, serum albumin 3.4 g/dL and proteinuria 3.03 g/24 h. On the account of the relevant proteinuria, she was started on a supplemented, low protein diet at the eighth gestational week.

**Figure 6:**
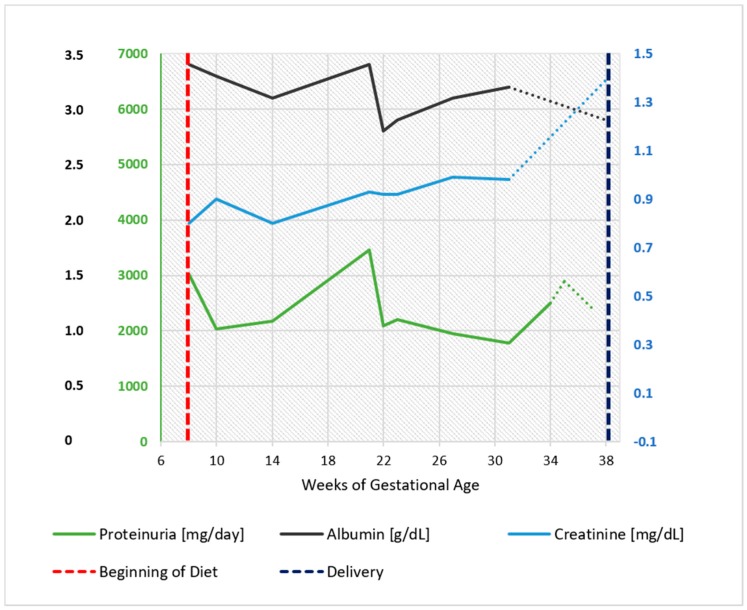
Case 2. Serum albumin, serum creatinine, and 24 h proteinuria during pregnancy and before and after the start of the diet. Biochemical data recorded in the last four weeks were collected in another center and are reported as dashed lines. Note: serum creatinine minimum: 0.85 mg/dL, maximum 0.95 mg/dL (GFR 70-80 mL/min).

The patient’s renal functional profile in pregnancy is reported in Figure 6, which shows that proteinuria and kidney function remained substantially stable throughout her pregnancy.

**Figure 7:**
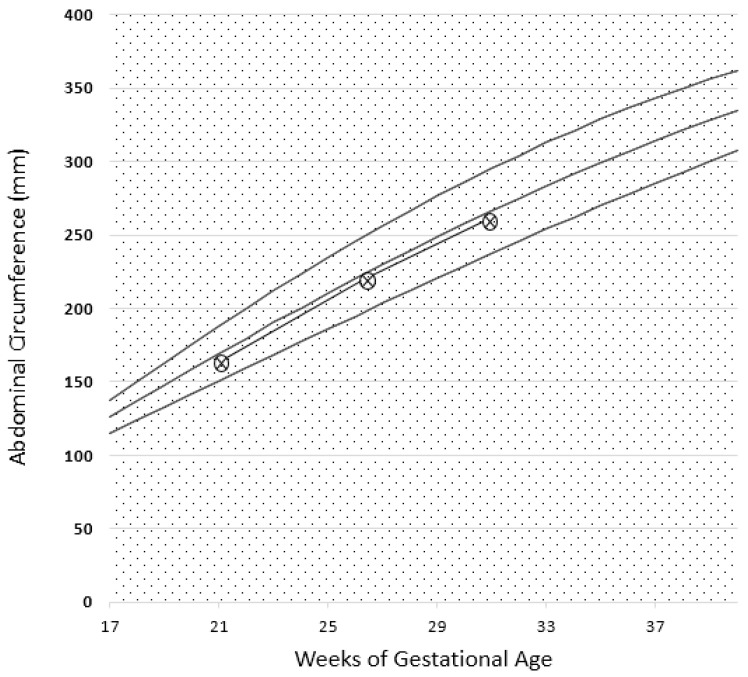
Intrauterine growth curve in Case 2. The parameter analysed is abdominal circumference (CA). Report based on SIEOG (Italian Society of Obstetric-Gynecological Ecography) Guidelines. Biometric curves: Paladini et al. .

The baby’s growth curve and arterial Doppler were normal (Figure 7). The patient was normotensive during pregnancy. She delivered at 38 gestational weeks and two days (spontaneous labor), giving birth to a healthy female baby, weighing 3300 g, with an Apgar index of 9 at the first min and 9 at the fifth min. Neonatal weight was in the 75th centile of the Italian neonatal weight curves (Ines Charts).

At the time of the present report, creatinine clearance was 84 mL/min, serum creatinine 1.1 mg/dL, and proteinuria 1.9 g/24 h. Treatment consists of 100 mg/day of cyclosporine and Ramipril. She is no longer following the diet. The baby, aged six months at the time of the present report, is developing normally.

### 2.3. Case 3

**Figure 8:**
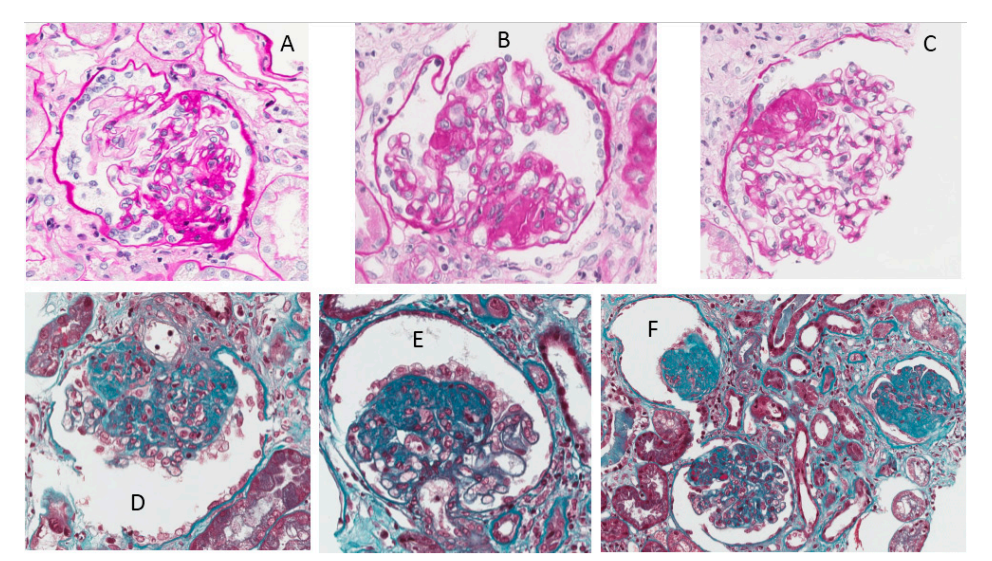
Kidney biopsy in Case 3. (**A**–**C**) Foci of segmental sclero-hyalinosis with consolidation of the glomerular tuft by an increased extracellular matrix, obliterating the glomerular capillary lumens. Sclerotic lesions are localized in perihilar and/or peripheral segments; (**A**) PAS original magnification 200×; (**B**) PAS original magnification 400×; (**C**) PAS original magnification 400×; (**D**,**E**) Segmental lesion of sclerosis with overlying, activated, hypertrophic, but not proliferating, podocytes, which form a ‘cap’. Occasional foam cells are entrapped in sclerotic lesions; (**F**) The central glomerulus shows peripheral segmental sclerosis; the right and the left glomerulus show global sclerosis. There is mild interstitial fibrosis and tubular atrophy; (**D**–**F**) Masson trichrome original magnification 400×.

This patient is an African woman, 39 years old at the time of the present report, with a histological diagnosis of focal segmental glomerulosclerosis (FSGS) linked to HIV infection six years previously (28 glomeruli, eight with global sclerosis, nine with segmental sclerosis and collapsed glomerular tuft; slightly widened, fibrous interstitium) (Figure 8). At the time of the kidney biopsy, which was performed shortly after the diagnosis of HIV infection, she presented a full-blown nephrotic syndrome with low-normal kidney function (serum creatinine 1 mg/dL, creatinine clearance on 24 h urine collection: 65 mL/min, serum albumin 2.1 g/dL, further decreased to a minimum of 1.3 g/day, proteinuria between 6 and 8 g/24 h). An attempt to employ steroid treatment was unsuccessful (proteinuria stable at around 9 g/24 h), and she was started on ACE inhibitors without substantial changes in proteinuria, but with a progressive increase in serum albumin. Conversely, antiviral therapy was successful and HIV-RNA rapidly became undetectable and remained so during follow-up, including during pregnancy.

At the start of her pregnancy, her treatment consisted in antiretroviral therapy, ACE inhibitors, which were discontinued, and low molecular weight heparin. Her biochemical data were as follows: creatinine clearance 142 mL/min, serum creatinine 0.84 mg/dL, serum albumin 3.23 g/dL and proteinuria 6.3 g/24 h. She was normotensive without therapy and remained so throughout pregnancy.

**Figure 9:**
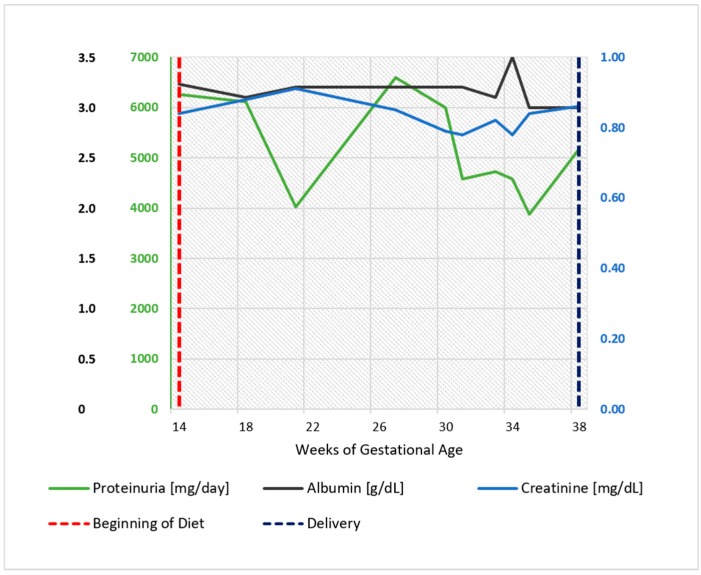
Case 3. Serum albumin, serum creatinine, and 24 h proteinuria during pregnancy and before and after the start of the diet. Note: serum creatinine minimum: 0.5 mg/dL at 22 weeks, maximum 0.9 mg/dL at 20 and 38 weeks.

**Figure 10:**
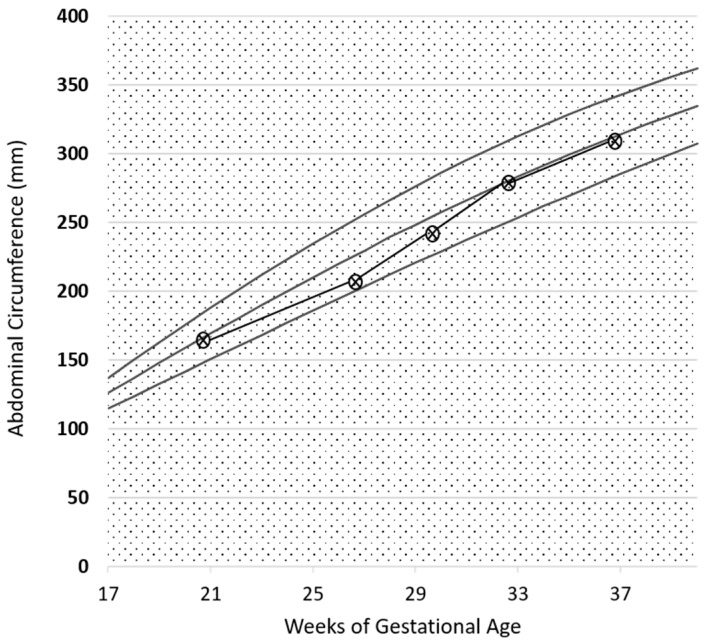
Intrauterine growth curve in Case 3. The parameter analysed is abdominal circumference (CA). Report based on SIEOG (Italian Society of Obstetric-Gynecological Ecography) Guidelines. Biometric curves: Paladini et al.

On account of the relevant proteinuria detected at referral, the patient was started on a supplemented, low-protein diet at the 14th week of gestation (Diet 3). The functional pattern is depicted in Figure 9. In her case too, proteinuria and kidney function remained substantially stable throughout her pregnancy. The baby grew on a harmonic, regular curve (Figure 10); uterine and umbilical arterial Doppler were normal.

The patient delivered a healthy male baby at 38 gestational weeks and one day (vaginal delivery; weight 2770 g, in the 18th centile according to the Italian reference standards, Apgar index 9 at the first min and 9 at fifth min). The patient discontinued the diet after delivery; she reported otherwise good health. The baby was developing normally. Six months after delivery, creatinine was 0.8 mg/dL, proteinuria 7 g/24 h, and HIV-RNA was not detectable.

## 3. The Diets

The indications and contraindications of low protein diets in pregnancy are debated as available data is scant. While some groups mitigate protein restrictions or suggest an unrestricted diet, ours has adapted moderately restricted, supplemented vegan-vegetarian low-protein diets, designed for patients with advanced CKD, to pregnant patients, increasing the protein intake from about 0.6 to about 0.8 g/kg/day and integrating the general nutritional indications on calories, calcium, and oligo-elements [26,27,28,30].

Since our diet is basically vegan but occasionally allows milk and yoghurt and at least one (and up to three) unrestricted meals per week, it was initially defined as ‘vegan-vegetarian’, but an alternative and possibly better term would be ‘plant based’, underlining the flexible diet approach based on, although not limited to, aliments of vegetal origin. We included supplementation with essential aminoacids and ketoacids to be ‘on the safe side’ for the intake of essential aminoacids, as has been described in detail elsewhere [26,27,28,30].

The basic prescriptions are as follows: protein intake 0.6–0.8 g/kg/body weight; no specific restriction in salt, potassium, or phosphate intake. Due to the vegan-vegetarian structure of these diets, potassium intake is usually high and phosphate is relatively low (on average 8 to 12 mg of phosphate in plant derived proteins, 30% to 50% absorbed). However, our decision was to limit the prescriptions to the quantity (0.6 to 0.8 g/kg/day) and the quality of proteins (plant-based diets, except for the unrestricted meals), without any further restriction, to ensure some flexibility and improve compliance [26,27,28,30] (see the actual diets in the appendices).

Iron and B12 are regularly controlled (every four to six weeks). Iron and folate are routinely supplemented in pregnancy and B12 only in the presence of low blood levels (not needed in the three cases here described).

In the absence of a validated nutritional assessment for CKD pregnancies, and since proteinuria impaired considering albumin, total proteins, and cholesterol as reliable nutritional markers, the nutritional evaluation was based on a dietary journal and dietary recall, assessed by an expert dietician.

The prescriptions of the diets changed over time; while the first diets were exclusively qualitative (no animal-derived food except in the unrestricted meals) and had no caloric restrictions (ad libitum diet: Appendix A.1 Diet 1), we further integrated them to prevent toxoplasmosis and listeriosis, and we limited the caloric intake, as well as salt and sugar intake (Appendix A.2 Diet 2). Moreover, as we felt it was important to adapt the diet to each patient, specific aliments of different ethnic origins were added in some cases (Appendix A.3 Diet 3).

A recent re-evaluation of the results obtained over the last 15 years allowed us to compare 36 on-diet CKD pregnancies with 47 control CKD cases on unrestricted diets. In spite of similar baseline conditions, the incidence of small for gestational age and/or extremely pre-term babies (<28th week) was significantly lower in singletons from on-diet mothers than in the controls, suggesting an effect of a ‘plant-based’ diet on the utero-placental circulation [28].

Such an effect could be due both to a decrease in ‘vaso-toxic’ elements and to an increase in ‘vaso-protective’ factors. In fact, plant-based diets reduce red-meat consumption, which is associated with an increase in cardiovascular risk and in oxidative stress; conversely, diets rich in vegetables, legumes, and grains may be protective against endothelial dysfunction [36,37,38,39,40,41,42]. A further specific advantage of vegetable proteins and of supplementation with ketoacids has been suggested in experimental models [43,44,45,46].

## 4. Discussion

A healthy diet during pregnancy is a key for the well-being of the mother and the fetus; in the western world, the concept of a healthy diet has shifted from protection from malnutrition to protection from overnutrition [47,48,49,50,51,52]. Mediterranean and plant-based diets may have a role in preventing many of the ‘overeating’ diseases, including metabolic syndrome and obesity [53,54]. At least in highly resourced countries, a well-balanced plant-based diet is safe and may protect from excessive weight gain in pregnancy [55,56].

The literature on plant-based diets in pregnancy is limited and heterogeneous. The position of the American Dietetic Association, “well-planned vegetarian diets are appropriate for individuals during all stages of the lifecycle, including pregnancy, lactation, infancy, childhood…”, is supported by a first systematic review based on seven papers [57]. A subsequent review, targeting plant-based diets outside the context of limited resources, analyzed 29 papers; none of the studies suggested a higher risk for adverse pregnancy-related events in vegan-vegetarian mothers [58,59,60,61,62,63,64,65]. The only relevant note of caution was the finding, in one large study, of a higher incidence of hypospadias in children from vegan mothers, which remained unexplained and thus unconfirmed [66]. Further important points are the avoidance of nutritional deficits, in particular of vitamin B12, vitamin D, and iron, all of which are regularly controlled in our on-diet patients [26,27,28]. A subsequent systematic review on zinc intake during pregnancy in women on plant-based diets suggested that this element should also be monitored [67].

Overall, therefore, these data supported our decision to offer a ‘plant-based option’ to CKD patients due to the potential advantages of controlling proteinuria and hyperfiltration [39,43,44,45,68,69,70].

The three cases described above, referred to in our unit from different settings and followed by different approaches before pregnancy, encompass three different facets of focal segmental glomerulosclerosis: ‘primary’ FSGS, in Case 1; forms associated with other diseases and considered a reflection of hyperfiltration on remnant nephrons in Case 2; and those associated with other autoimmune derangements, including AIDS, in Case 3.

Within the limitations of sporadic experiences, these three cases, one of which was included in previous publications (Case 1, 27, 28), further underline the nutritional safety of a moderately protein restricted, supplemented, plant-based diet in pregnancy. The qualitative approach, elsewhere described in detail and examples of which are available in the appendix, is adaptable to different dietary habits, as shown by the inclusion of traditional Italian or African foods in the diets designed for Patients 2 and 3 (appendices).

In the absence of validated nutritional markers in CKD pregnancies, we considered that the stability of serum albumin, in spite of the reduction in protein intake, was in keeping with a positive effect of our low-protein diets on protein balance [43,68,69,70,71,72,73,74,75,76,77,78].

Proteinuria often increases in gestation in patients with glomerular diseases; however, this increase was not observed in our patients (Figure 1, Figure 2, Figure 6, Figure 7, Figure 9 and Figure 10). Interestingly, none of the patients developed a rapid reduction in renal function after delivery, which was different from many cases in the literature [79,80,81,82,83,84,85,86].

Furthermore, we would like to comment on the particular pattern seen in Case 1; the woman with a diagnosis of ‘primary FSGF’ had refractory proteinuria, which had only partially responded to a vast array of treatments. Not only was her proteinuria at start of her pregnancy higher than at the end of her pregnancy, but also, when she resumed a normal protein diet, this was followed by a marked increase in proteinuria and then followed by a remission after restarting the vegan-supplemented diet. While this pattern cannot be fully explained and spontaneous remission of the nephrotic syndrome in FSGS has occasionally been reported, the temporal relationship between diet and proteinuria, in the context of stable kidney function, may point to the presence of alimentary antigens (or of deep changes in the microbiota) in the pathogenesis of her disease, as described in the past, mainly in children, and more recently also in experimental animals. The allowance of gluten in our patient may point towards a role of dairy products (Figure 1 and Figure 3) [87,88,89,90,91,92,93,94].

## 5. Conclusions

In summary, our report, within the limit of a small, non-controlled case series of three cases only, suggests that a moderately protein-restricted, supplemented, plant-based diet might contribute to controlling proteinuria in pregnant women affected by FSGS. While the rarity of the combination of pregnancy, glomerular sclerosis, preserved kidney function, relevant proteinuria, and good nutritional status makes it infeasible to conduct a randomized trial (if such a trial was to be deemed ethically acceptable), this approach may respond to the imperative of ‘primum non nocere’ and may offer an alternative to the therapeutic nihilism in these proteinuric nephropathies in pregnancy. However, further studies are warranted to confirm the potential value of such a treatment strategy.

## Appendix A. The Diets

### Appendix A.1. Diet 1

#### **A** **‘vegan/vegetarian’ diet supplemented with alpha-keto analogues**

This diet is based on the combination of a vegan-vegetarian diet (no animal derived food), supplemented with essential aminoacids and ketoacids to protect from malnutrition.

**Important:** There are very few studies in the medical literature on low-protein diets in pregnant CKD patients and our experience, obtained from a limited number of patients, is one of the few available ones. To date, the results have been highly positive, but you should report any doubts or inform us if you have side effects to help us follow you better and understand what we can do to improve our approach.

In short:

You can eat anything that grows on the earth, under the earth, and on trees and anything that is derived from what grows over and under the earth and on trees (plants). The only limit is your weight; be careful with fruit, as fruit is rich in sugar and sugar is allowed ‘in moderation’ in pregnancy. Olive oil, which is derived from trees and has no sugar, can be used freely.

You should not eat anything that walks on the earth, flies in the air, or swims in the water or anything that is derived from animals, with the exception of butter, which contains mainly protein-free fat. However, your use of butter should also be limited (not too much fat in pregnancy).

However, in the ‘free’ meals you can eat anything you want (quality) but not too much (quantity) to avoid gaining too much weight.

Supplements (Alfa-Kappa or Ketosteril) are prescribed as one tablet for every 8-10 kg of body weight per day, taken with your main meals. The tablets can be taken during the meal or at the beginning or end of the meal. The number of pills may increase (or decrease) in pregnancy depending on your condition and biochemical test results.

In Detail:

Breakfast:

For breakfast, you can have tea or coffee with bread or biscuits with jam, cereal (such as corn flakes), or a slice of homemade cake (if you cannot do without it, you are allowed to have milk at breakfast).

Alternatively, you can have bread with olive oil and tomatoes or olives, or crackers or bread sticks made with extra virgin olive oil.

One or two (specify) tablets of alpha-kappa.

Lunch and Dinner (for each meal), please combine the following:

Pasta, rice, couscous, or cereals (barley, millet, kamut, wheat) dressed with oil, preferably olive oil, and accompanied by:

- Legumes (chickpeas, peas, beans, lentils, etc.).
- Vegetables of any kind (raw or cooked) (see indications for toxoplasmosis).
- Bread, bread sticks, or crackers with made with extra virgin olive oil.
- Fresh fruit (indicatively 150–200 g).

Two or three (specify) tablets of alpha-keto analogue.

Potatoes can replace bread or pasta.

Dressings/Cooking Methods:

Food can be cooked as you prefer (stewed, steamed, grilled, broiled, baked, sautéed in extra virgin olive oil).

Use extra virgin olive oil for seasoning and avoid mixed seed oil, butter, lard, margarine, cream, and sauces.

There are no limitations on the use of natural spices, herbs (rosemary, sage, basil, oregano, thyme, parsley), hot peppers, onion, garlic, lemon juice, vinegar, or balsamic vinegar.

Drinks and Sweets:

Drink water (still or sparkling) throughout the day.

Wine and beer should only be drunk occasionally and in small quantities.

### Appendix A.2. Diet 2 (Modified from Reference [28])

#### **A** **‘Vegan/Vegetarian’ Diet For Pregnant Women with CKD (Moderate Protein Restriction, Supplemented with Alpha-Keto Analogues)**

This diet is based on some very simple assumptions:

- a low-protein diet is associated with a reduction in the functional ‘workload’ of the diseased kidneys; this is important since kidneys need to ‘work harder’ during pregnancy, which may increase proteinuria and thus reduce kidney function in the long term.
- proteins of vegetable origin induce a smaller ‘workload’ for the kidneys than animal-derived proteins do, and therefore a ‘vegan-vegetarian’ diet is better suited to stabilizing renal function in pregnant women with chronic kidney disease.
- vegan (no animal or animal-derived food of any kind) and vegetarian (no food from a living source; eggs, milk, and derivates are allowed) diets are safe in pregnancy (regardless of the presence of CKD), if well balanced and controlled for protein deficiencies.
- the diet is compatible with a good quality of life and has to be followed with flexibility, adapting to the preferences of the individual. In pregnancy, we need to pay more attention to maintaining an adequate level of several nutrients, including vitamins and minerals (the most important ones that we know are vitamin B12, folic acid, vitamin D, and calcium), and to not gain too much weight (this is why we ask you to pay attention not only to the quality of the food you eat, but also to the quantity).
- proteins are contained in ‘animal-derived’ food (meat, fish, eggs, poultry, and dairy products) and in plant-derived food (grains, cereals, legumes, soya, etc.). While some animals (cows, for example) are able to build up all the amino acids from ‘energy’ (i.e., grass), humans are not. Therefore, ‘animal-derived’ proteins are called ‘noble’ or ‘complete’ since they contain all the protein components (amino-acids) that we need; plant-derived proteins are called ‘incomplete’ or ‘non noble’ since their proteins do not contain all the amino acids we need. Every plant-derived protein contains some of them, but we need to put together several different types of plant-derived food to complete them.
- the use of ‘supplements’ (called alpha-kappa or ketosteril, which are mixtures of essential amino acids), allows ‘completion’ of the vegetable proteins, avoiding the risk of nutritional deficits even in patients who do not have time to combine different ‘plant-derived’ foods.

In short: (see previous diet)

In Detail:

Breakfast:

For breakfast you can have tea, coffee, a soy drink or soy yoghurt, with bread or biscuits with jam, cereal (such as corn or oat flakes or muesli), or a slice of homemade cake (butter, oil, egg yolk, and small quantities of milk or yoghurt are allowed). If you cannot do without it, you are allowed to have milk at breakfast.

Alternatively, you can have bread with olive oil and tomatoes or olives, bread and tofu, or crackers or bread sticks with extra virgin olive oil.

One or two (specify) tablets of alpha-kappa.

Lunch and Dinner (for each meal), please combine the following:

- Pasta, rice, couscous, or cereals (barley, millet, kamut, wheat) dressed with oil, preferably olive oil, and accompanied by two different vegetables and two different legumes at each meal.
- Legumes (chickpeas, peas, beans, lentils).
- Vegetables of any kind (raw or cooked) (see indications for toxoplasmosis).
- Bread or bread sticks or crackers made with extra virgin olive oil.
- Fresh fruit (indicatively 150–200 g).

Two or three (specify) tablets of alpha-keto analogue.

Potatoes can replace bread or pasta.

Legumes should be consumed in at least one main meal in association with pasta, rice, or another cereals. If you like, you can use tofu, tempeh, or seitan instead of legumes.

Oily nuts such as walnuts (four to six per day) are useful for their high content of ‘good’ fat like olive oil, and they protect from atherosclerosis and help protect the placental vessels.

Snacks:

Snacks (mid-morning, mid-afternoon) are welcome:

- 1 cup of soy yoghurt or soy drink.
- Bread, biscuits, crackers, or bread sticks made with extra virgin olive oil.
- Bread with tofu and olives and olive paste, tomato, vegetables.
- 1 cup of plain low-fat yoghurt.
- 1 piece of fresh fruit.
- raw vegetables like fennel, celery, peppers, cucumbers, tomatoes, and carrots may be used if you are not receptive to toxoplasmosis (wash very well in any case).
- (regular yoghurt can be used occasionally).
- for this patient, who was of Sicilian origin, the snacks were identified as toasted chickpeas (15% protein content) and lupins (12% to 15% proteins), both rich in iron, typical of Sicilian cuisine and easily found in her setting.

Dressings/Cooking Methods:

Food can be cooked as you prefer (stewed, steamed, grilled, broiled, baked, sautéed in extra virgin olive oil).

Use extra virgin olive oil for seasoning and avoid mixed seed oils, butter, lard, margarine, cream, and sauces (such as mayonnaise, ketchup, tuna sauce, etc.) and items containing sodium glutamate (not good for your blood vessels or for the placental vessels). Also avoid vegetable fat, non-hydrogenated vegetable fats, palm oil, or coconut oil (similar reasons).

There are no limitations on the use of natural spices, herbs (rosemary, sage, basil, oregano, thyme, parsley), hot peppers, onion, garlic, lemon juice, vinegar, balsamic vinegar, miso, tamari, or shoyu; if using tamari or miso, you should check to see that they do not have added ingredients.

Use iodized salt, which should not be confused with ‘sea salt’ or ‘whole salt’. Iodized salt is common salt to which iodine has been added (good for thyroid function). In order to increase the intake of useful minerals (calcium, iron, potassium) about one tablespoon of sesame seeds, sunflower seeds, or pumpkin seeds may be added to foods. You should eat baked goods (crackers, bread sticks) without added fat or made with extra virgin olive oil, sunflower, or corn oil.

Drinks and Sweets:

Drink water (still or sparkling) throughout the day. Check with your nephrologist to establish the amount. Avoid wine and beer.

Cocktails, spirits, and liqueurs are forbidden. Due to their high sugar content, avoid soft drinks, syrups, juices, fruit juices, and soluble herbal tea.

Reduce your intake of foods with high sugar content; brown sugar, ice cream, honey, malt, fruit jellies, croissants, cakes, cream, chocolate, cookies, candy, etc. Avoid artificial sweeteners like aspartame (E951), acesulfame K (E950), saccharin (E954), Sucralose (E955), cyclamate (E952), and neohesperidine DC (E959).

Toxoplasmosis and Listeriosis (two infections that may be transmitted by food):

If you are receptive to toxoplasmosis, eat only well-cooked vegetables and meat (during the free meals).

Be especially careful that strawberries and other berries, mushrooms, and fresh herbs (parsley, basil, sage) are thoroughly washed.

To prevent listeriosis, avoid vacuum-packed products (such as smoked salmon), raw milk, and unpasteurized cheese (such as gorgonzola, taleggio).

### Appendix A.3. Diet 3 (Same as Diet 2, but African Dishes Corresponding to the Patent’s Habits Were Added)

An Example: A Day:

Breakfast:

You may have tea or coffee with a small amount of milk or a soy drink enriched with calcium or soy yogurt in association with bread or biscuits with jam, cereal (such as corn or oat flakes or muesli), or a slice of homemade cake (small amounts of butter, oil, egg yolk, milk, or yoghurt are allowed). Alternatively, you can have bread with olive oil and tomatoes; olives, bread, and tofu; or crackers or bread sticks made with extra virgin olive oil.

Two tablets of alpha-keto analogue.

Lunch and Dinner:

At lunch, it is important to eat:

- A portion of pasta, rice, couscous or cereal (barley, millet, kamut, wheat), fufu/garri, yams, tapioca, or plantain, dressed with vegetables, tomato, garlic, and olive oil.
- You can have a portion of legumes (chickpeas, peas, beans, lentils) or moimoi without meat or fish
- Vegetables of any kind (raw or cooked) can be included (see indications for toxoplasmosis)
- Lunch can also include a serving of bread, bread sticks, crackers made with extra virgin olive oil and a serving of fresh fruit (indicatively 150–200 g).

Two tablets of alpha-keto analogue.

Potatoes can be eaten instead of bread or pasta. Legumes should be consumed in at least one main meal (lunch or dinner) in association with pasta, rice, cereal, or tapioca. If preferred, vegetable proteins such as tofu, tempeh, or seitan can be eaten instead of legumes.

Snacks

Snacks (mid-morning, mid-afternoon) are welcome:

- one cup of yoghurt, one cup of soy, or a soy drink enriched with calcium.
- Bread, biscuits, crackers, or bread sticks made with extra virgin olive oil.
- Bread with tofu or olives and olive paste, tomato, and vegetables.
- one cup of plain low-fat yogurt and one serving of fresh fruit.

Oily nuts such as walnuts (four to six per day) are allowed and are useful for their high content of omega 3. Peanut butter may be added to dressings and sauces.
